# miR-320 Regulates Glucose-Induced Gene Expression in Diabetes

**DOI:** 10.5402/2012/549875

**Published:** 2012-07-31

**Authors:** Biao Feng, Subrata Chakrabarti

**Affiliations:** ^1^Department of Pathology, Western University, London, ON, Canada N6A 5C1; ^2^London Health Science Centre, 339 Windermere Road, London, ON, Canada N6A 5A5

## Abstract

miRNAs play an important role in several biological processes. Here, we investigated miR-320 in glucose-induced augmented production of vasoactive factors and extracellular matrix (ECM) proteins. High glucose exposure decreased the expression of microRNA 320 (miR-320) but increased the expression of endothelin 1 (ET-1), vascular endothelial growth factor (VEGF), and fibronectin (FN) in human umbilical vein endothelial cells (HUVECs). Transfection of miR-320 mimics restored ET-1, VEGF and FN mRNA, and protein expression in HUVECs treated with high glucose. Furthermore, miR-320 mimic transfection reduced glucose-induced augmented production of ERK1/2. Data from this study indicates that miR-320 negatively regulates expression of ET-1, VEGF, and FN through ERK 1/2. Identification of such novel glucose-induced mechanism regulating gene expression may offer a new strategy for the treatment of diabetic complications.

## 1. Introduction

microRNA- (miRNA-) mediated regulation of gene expression has emerged as a major mechanism in regulating basal and stress-induced alterations of gene expression in several diseases [1–5]. miRNAs are endogenous, single-stranded, nonprotein coding RNAs, approximately 19–24 nucleotides in length, that are major posttranscriptional regulator of RNA [6, 7]. Most miRNAs negatively regulate gene expression at the posttranscriptional level, by interacting with their target mRNA 3′ untranslated region (UTR) [8]. Mature miRNA sequences are also highly conserved among species. Interestingly, based largely on bioinformatics analyses, one miRNA may regulate a large number of target genes, and a single gene may also be regulated by multiple miRNAs, targeting various sites of the gene [9]. They have been implicated as players in multiple biologic processes such as differentiation, proliferation, apoptosis, and in providing feedback loops for various signal transduction pathways [7, 9].

 Recent data from several laboratories have emerged with respect to miRNA alterations in diabetic complications [10–12]. We have shown that miRNA-mediated gene regulation in diabetes interacts with other epigenetic changes such as histone acetylation [10, 11]. We and others have demonstrated alterations of miR-133a in cardiomyocyte hypertrophy in diabetes [11, 13]. We have recently demonstrated that miR-200b regulates VEGF and control permeability and angiogenesis in diabetic retinopathy [10]. We have also shown that increased fibronectin (FN) production in chronic diabetes is regulated by miR-146a [11].

In diabetes, glucose induced augmented expression of vasoactive factors and ECM proteins are important mechanisms causing tissue damage [14]. Several investigators have demonstrated augmented expression of vasoactive factors such as endothelin-1 (ET-1), vascular endothelial growth factor (VEGF), and extracellular matrix (ECM) proteins such as FN in all organs, affected by chronic diabetic complications [10, 11, 14, 15]. Oxidant injury in hyperglycemia is known to activate multiple signalling pathways which in turn lead to increased expression of several such molecules [16]. Such signalling pathways include activation of mitogen-associated protein kinases (MAPK) [17, 18]. We and others have shown that extracellular signal-regulated kinase ERK 1/2 activation can be regulated by protein kinase C (PKC) [17, 19]. In addition, diabetes-induced ET-1 upregulation can also cause MAPK activation [19]. Hence, it appears that several interactive molecules and signalling pathways may contribute to alteration of the effector vasoactive factors and ECM proteins in diabetes. As miRNAs control expression of almost all biological molecules, it is possible that several of these factors are also posttranscriptionally controlled by specific miRNAs. Furthermore, as one gene may be regulated by multiple miRNA, it is potentially possible to modulate transcription of multiple genes using a single miRNA.

In this study, we focused on a specific miRNA, that is, miR-320, which demonstrated glucose-induced downregulation. miR-320 has been found to have widespread biological effects as it regulates multiple important molecules. Its potential targets include ET-1, ERK1, VEGF, and FN (http://www.microrna.org/). Its biologic actions include effects on carcinogenesis, development, and ischemia reperfusion injury [20]. In a recent study, serum level of miR-320 was found to be reduced in the diabetic population [21]. On the other hand, increased miR-320 has been demonstrated in the cardiac microvascular cells in the diabetic rats [22]. As endothelial cells are primary targets in diabetic complications, we used a well-established Human Umbilical Vein Endothelial Cells (HUVECs) for these studies. We and others have demonstrated glucose-induced alterations of the multiple molecules in these cells [10, 11, 19].

## 2. Materials and Methods

### 2.1. Cell Culture and Treatments

All reagents were purchased from Sigma (Oakville, ON, Canada) unless specified. HUVECs were obtained from Bio-Whittaker (San Diego, CA, USA) and plated in complete endothelial growth medium supplemented with 5% fetal bovine serum, endothelial cell growth supplement (Bio-Whittaker), and 100 ug/mL penicillin/streptomycin. Cells were plated at a density of 1 × 10^5^ cells/mL. They were treated with glucose (25 mmol/L). After 24 h in serum-free, the cells were transfected with miRIDIAN micro RNA Mimic miR-320 (100 nM) (DHARMACON Inc., Chicago, IL, USA) using transfection reagent Lipofectamin, 2000 (Invitrogen, ON, Canada). Negative control miRNA, (100 nM) were used for control transfection.

### 2.2. Microarray Analysis for miRNA Expression

Total miRNAs were extracted from HUVECs using the mirVana miRNA isolation kit (Ambion, Inc., Austin, TX, USA), according to the manufacturer's instruction. Briefly, the cells were collected and lysated in the Lysis/Binding solution. miRNAs additive (1 : 10) were added to the lysate and incubated for 10 minutes on ice. And equal volume acid-phenol : chloroform was added to the lysate. Following centrifugation and removal of the aqueous phase, the ethanol was added into the mixture. The mixture was passed through the filter cartridge and was eluted with elution solution.

miRNA expression profiling was performed following the protocol provided by the manufacturer (Life Technologies, ON, Canada). In brief, 1 ug of isolated total miRNAs was added with Megaplex RT primer, dNTPs, MultiScribe reverse transcriptase, RNase inhibitor, and buffer to perform megaplex reverse transcription. Following reverse transcription, the real-time PCR reactions mixture was prepared to mix the Megaplex RT product with TaqMan universal pCR master mix, No AmpErase UNG (2x). The real-time PCR reactions mixture was dispensed into each port of the TaqMan microRNA array card to run the array using the 384-well TaqMan low-density array default thermal cycling conditions in the 7900HT system (life Technologies, ON, Canada). The results were shown using relative quantification (ΔΔCt).

### 2.3. miRNA Validation by Real-Time RT-PCR

miRNAs were isolated using mirVana RT-PCR miRNA isolation kit. RT was performed after isolating of miRNA (Ambion Inc., Austin, TX, USA). TaqMan real-time PCR was used to analyze the expression of miR-320 by using miR-320 assay (Ambion Inc., Austin, TX, USA) following manufacture's instruction. The data were normalized to U6 snRNA to account for differences in reverse-transcription efficiencies and amount of template in the reaction mixtures.

### 2.4. Real-Time RT-PCR for mRNA

RNA was extracted with TRIzol reagent (Invitrogen Canada Inc., ON, Canada) as previously described (total RNA (2 *μ*g), was used for cDNA synthesis with cDNA reverse-transcription kit (Applied Biosystems Inc., CA, USA). Real-time quantitative RT-PCR was performed using the LightCycler as described [10, 11] (Roche Diagnostics Canada, QC, Canada). The primer sequences have been listed in Table 1. For more details about *β*-actin, see Table 1. *β*-actin mRNA was used as an internal control.

### 2.5. Western Blotting

The cells were washed with PBS and lysed with ice-cold RIPA lysis buffer (Upstate, Temecula, CA, USA). Cell lysates were loaded to 10% SDS-PAGE gel and blotted with high-affinity rabbit polyclonal anti-ERK1/2 antibody (Cell Signaling Technology Inc., Danvers, MA, USA) followed by incubation with goat antirabbit secondary IgG antibody with horseradish peroxidase conjugate (Santa Cruz Biotechnology, Inc., CA, USA) using 1 : 5000 dilutions. Immunopositive bands were visualized with enhanced chemiluminescence advance Western blot detection system (Amersham Biosciences, Piscataway, NJ, USA). Blots were stripped and reprobed with *β*-actin (1 : 2000) as an internal control.

### 2.6. ELISA for ET-1 and FN Expression

Supernatants were collected from HUVECs cultured for FN, ET-1, and VEGF detection by ELISA. ELISA for FN was performed using a commercially available kit (Abcam, Cambridge, MA, USA) according to the manufacturer's instructions. Similarly, ET-1 and VEGF were measured using specific ELISA kit (Biomedica Medizinprodukte GmbH & Co KG, Wien, Austria) following the manufacturer's instructions.

### 2.7. Statistical Analysis

All experimental data are expressed as means ± SE and were analyzed by ANOVA or Student *t*-test as appropriate. A *P* value of 0.05 or less was considered significant.

## 3. Results

### 3.1. miR-320 Is Downregulated in the Endothelial Cells (ECs) in Response to Elevated Glucose Levels

Hyperglycemia is a key initiating factor for endothelial damage in diabetes. To identify underlying mechanisms of tissue damage, we initially analyzed miRNAs in ECs exposed to 5 mM (simulating euglycemia, LG) and 25 mM (simulating hyperglycemia, HG) glucose, using an miRNA PCR-array. Twenty-five mM glucose exposure for 24 hrs caused significant downregulation of 20 miRNAs and upregulation of 24 miRNAs.

**Figure 1:**
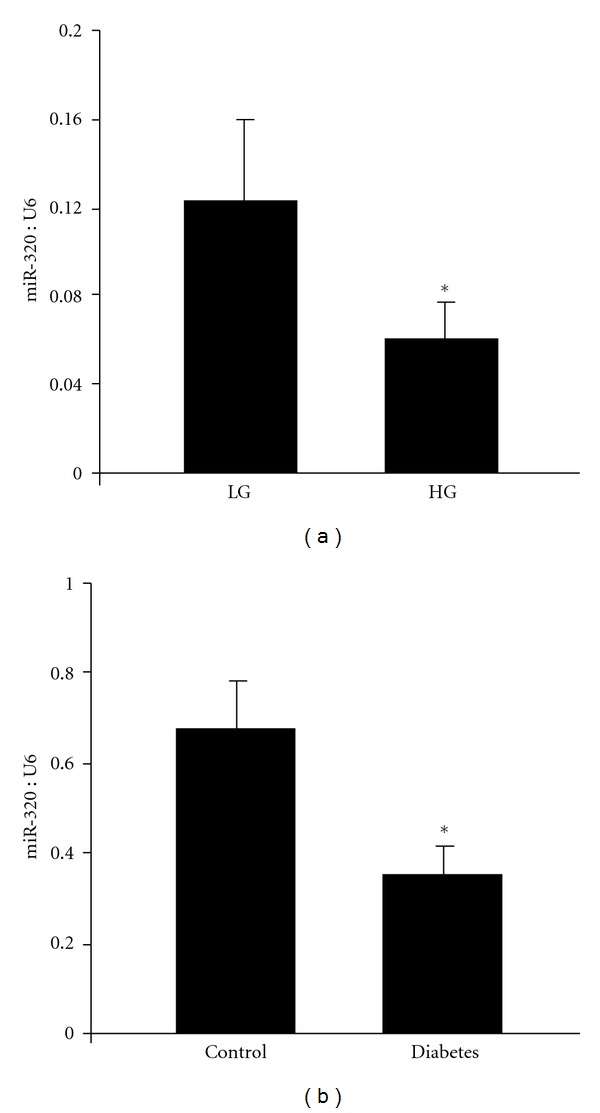
(a) 25 mM D-glucose (HG) caused decreased miRNA-320 expression in the endothelial cells compared to 5 mM D-glucose (LG) by real-time RT-PCR. (b) Similarly, diabetes caused reduced miR-320 expression in the kidney. (miRNA expressed as a ratio of U6 snRNA (U6). *P < 0.05 compared to LG or control; *n* = 6/treatment; analyses were performed using mirVana miRNA isolation kit and TaqMan microRNA assay system).

Using open-sourced softwares (http://www.targetscan.org/, http://www.microrna.org/, http://www.ebi.ac.uk/) for miRNA target predictions, miR-320 (identified to be significantly downregulated in the endothelial cells exposed to high glucose) was found to be associated with ET-1, VEGF, and FN. We verified the downregulation of miR-320 with qRT-PCR in the ECs (Figure 1(a)). No change in miR-320 level was observed when the cells were challenged with 25 mM L-glucose (osmotic control).

### 3.2. miR-320 Is Downregulated in the Kidneys of Diabetic Animals

We then wanted to see whether such changes are indeed of significance in a clinically relevant model of diabetic complication. To this extent, we examined renal cortical tissues from STZ diabetic rats, one month after onset of diabetes, as increased ECM proteins along with augmented ET-1 and VEGF production are known to occur in diabetic nephropathy. Diabetic animals showed hyperglycemia, glucosuria, and reduced body weight gain (data not shown). Analyses of miR-320 levels demonstrated a significantly reduced miR-320 level in the renal cortex of these animals (Figure 1(b)).

### 3.3. miR-320 Regulates Glucose-Induced Upregulation of Vasoactive Factors and ECM Protein in the Endothelial Cells

To establish a cause-effect relationship, we again used HUVECs as an *in vitro* model system. As previously mentioned, we and others have shown that endothelial cells exposed to high levels of glucose (simulating hyperglycemia) recapitulate molecular and functional features of diabetic vascular pathologies including upregulation of FN, ET-1, and VEGF [10, 11, 19].

**Figure 2:**
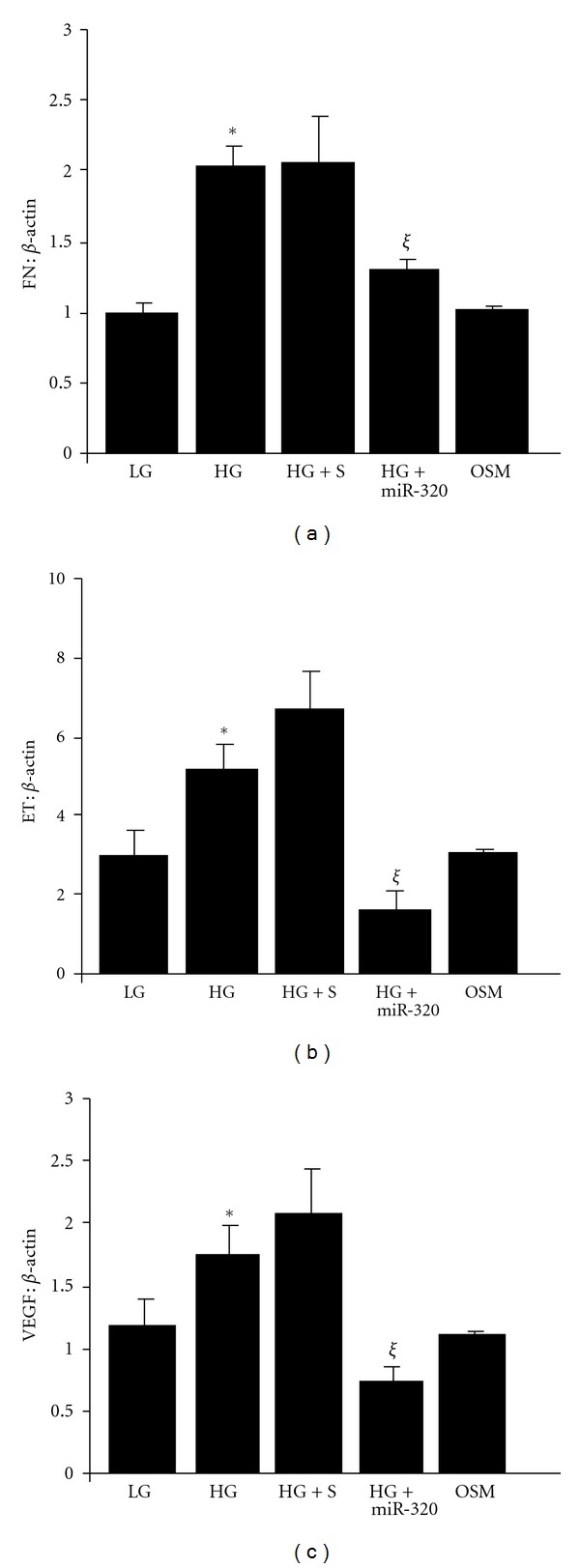
HG caused increased (a) FN mRNA, (b) ET-1, and (c) VEGF expression by real-time RT-PCR in the HUVEC. HG-induced increased FN, ET-1, or VEGF mRNA expression was prevented following miR-320 mimic transfection. But not by scrambled (S) mimic transfection (HG: 25 mM D-glucose, LG: 5 mM D-glucose., *P < 0.05 compared to LG, mRNA levels expressed as a ratio to *β*-actin. OSM: 25 mM L-glucose, *ξ* = *P* < 0.05 compared to HG).

 In parallel to decreased miR-320 level upon exposure to HG, mRNA levels of ET-1 and VEGF (measured by qRT-PCR) were increased. Such increases were prevented by miR-320 mimic transfection (Figure 2). Transfection efficiencies, assessed by measuring miR-320, expression showed >10-fold increase in intracellular miR-320 expression compared to scrambled miRNA transfection. We did similar experiments with ECM matrix protein FN. Glucose-induced FN upregulation in the HUVECs was significantly prevented by miR-320 mimic transfection (Figure 2).

**Figure 3:**
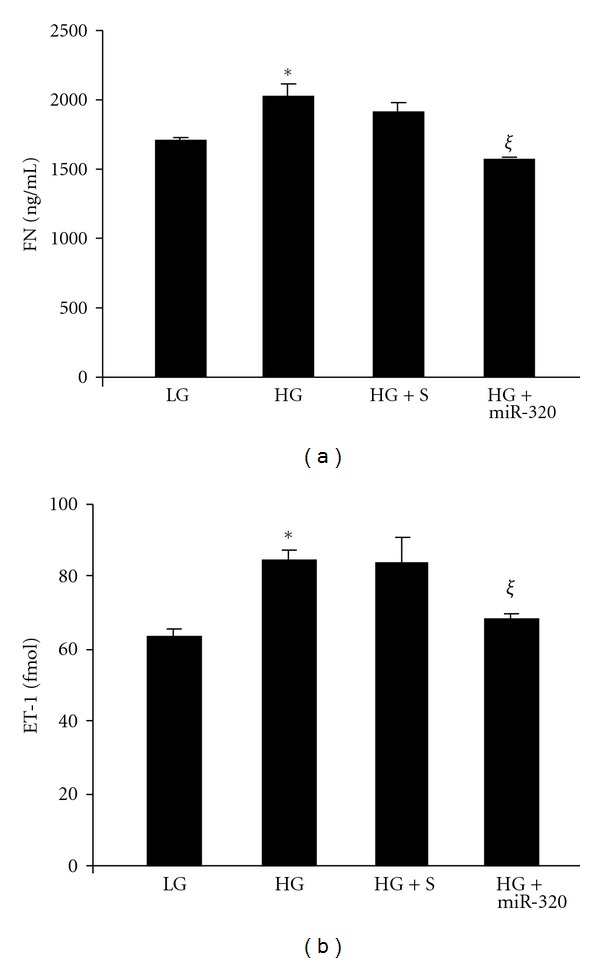
HUVECs exposed to 25 mM glucose (HG) compared to 5 mM glucose (LG) showed (a) increased FN protein and (b) increased in ET-1 protein. Transfection of endothelial cells with miR-320 mimics (but not the scrambled mimics) reduced glucose induced upregulation of FN and ET-1 protein levels. (S: scrambled miRNA, miR-320: miR-320 mimic, *significantly different from LG, *ξ*: significantly different from HG).

As microRNAs are posttranslational modifiers, we further examined protein levels of FN and ET-1 using ELISA. In keeping with RNA data miR-320 transfection prevented glucose induced upregulation of FN and ET-1. No effects were seen when cells were transfected with scrambled mimic (Figure 3).

### 3.4. miR-320 Regulates ERK1/2

**Figure 4:**
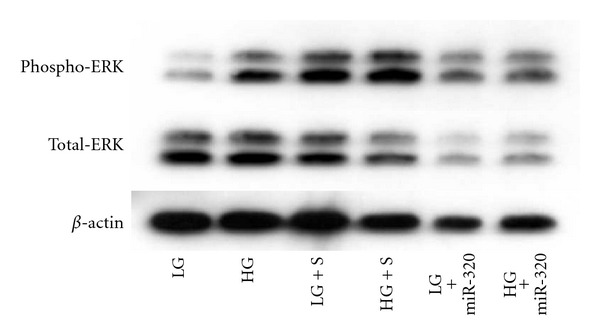
25 mM glucose (HG) caused increased expression of total ERK1/2 (ERK) and phosph-ERK1/2 proteins in the HUVECs, compared to 5 mM glucose (LG). Such increases were prevented by miR-320 mimic transfect ion. (S = scrambled mimics).

As mentioned earlier, ERK1/2 activation plays a significant role in increased vasoactive factor and ECM protein production in diabetes through PKC activation or through ET-1. Furthermore, miR-320 targets ERK. Hence, we used Western blot using antibodies against total and Phospho-ERK. miR-320 mimic transfection significantly reduced ERK1/2 protein levels and glucose induced ERK1/2 phosphorylation (Figure 4).

## 4. Discussion

In this study, we demonstrated that miR-320 is downregulated following glucose exposure to the endothelial cells and in the kidneys of diabetic rats. We have further showed that glucose induced upregulation of multiple vasoactive factors and extracellular matrix protein FN are regulated by miR-320. We also demonstrated that such regulations possibly work through MAPK modulation.

Following initial identification by a PCR-based microarray, we validated glucose induced downregulation of miR-320 in the endothelial cells. We also found that such downregulation is present in the kidneys of diabetic rats. We then demonstrated functional significance of these changes at the mRNA and protein levels using miR mimic transfection and normalization of glucose-induced upregulation of specific transcript. Our data indicated a role of ERK1/2 in this process. We used HUVECs to identify the* in vitro* biologic significance, which is a well-studied cell system in vascular biology, including the study of diabetic complications [10, 11, 19]. Although miRNA320 reduction has been demonstrated in the serum of diabetic individual and in the cardiac microvascular cells of diabetic rodents, this is the first study that demonstrated functional significance of such changes [22]. This study also demonstrates a potential therapeutic implication of miR-320 in chronic diabetic complications as it is able to modulate multiple transcripts.

miRNAs highly conserved molecules across the species. They are produced as small, nonprotein coding RNAs and mostly negatively regulate gene expression at the posttranscriptional level by interacting with their target mRNA 3′ untranslated region (UTR) [1, 5]. Most target mRNA predictions for miRNAs stem from computational analysis examining sequence complementarity [6, 7].

As we start to understand functions of various miRNAs, with their widespread role in biological processes, several of them appear to play significant roles in chronic diabetic complications. We have previously demonstrated the role of miR-133a in diabetic cardiomyopathy [23]. Other investigators have shown the role of miR-192 in diabetic nephropathy [13]. We have also recently demonstrated downregulation of miR200b and 146a in several chronic diabetic complications [10, 11]. The mechanisms by which hyperglycemia causes cellular damage in the context of chronic diabetic complications are indeed complex and are not fully understood. However, it is now accepted that glucose-induced oxidative stress plays an important role [16]. Such oxidative stress causes DNA damage and modifies transcription machinery through the activation of the redoxsensitive transcription factors [16]. Increased oxidative stress causes altered expression of a number of genes including VEGF, ET-1, and FN [24]. All such transcripts may, however, be post translationally regulated by miRNAs. Hence, miRNAs lend themselves to be therapeutic agents. However, the challenge for miRNA research is to define the function of specific miRNAs in various tissues and in the context of specific disease state.

 In summary, we have identified a specific miRNA, that is miR-320 in the endothelial cells exposed to high glucose. We have also demonstrated that miR-320 is important in the regulation of several transcripts of interest in chronic diabetic complications. Understanding such novel pathways will help to better understand pathogenesis of chronic diabetic complications and pave the pathways toward the development of novel adjuvant treatment.

## Figures and Tables

**Table 1:** Oligonucleotide sequences for RT-PCR.

Gene	Sequence (5^′^ → 3^′^)
ET-1	5^′^AAGCCCTCCAGAGAGCGTTAT3^′^
	5^′^CGAAGGTCTGTCACCAATGT3^′^
	6FAM-TGACCCACAACCGAG-GBNFQ

FN-1	5^′^GATAAATCAACAGTGGGAGC3^′^
	5^′^CCCAGATCATGGAGTCTTTA3^′^

VEGF	5^′^TCCTCACACCATTGAAACCA3^′^
	5^′^GATCCTGCCCTGTCTCTCTG3^′^

*β*-actin	5^′^CATCGTACTCCTGCTTGCTG3^′^
	5^′^CCTCTATGCCAACACAGTGC3^′^
